# Dietary supplementation with D-ribose enhances growth performance, improves serum antioxidant capacity, and inhibits rumen microbial LuxS/AI-2 quorum sensing of Hu sheep

**DOI:** 10.5713/ab.25.0291

**Published:** 2025-10-22

**Authors:** Jing Ge, Yanjiao Li, Xianghui Zhao, Kehui Ouyang, Mingren Qu, Qinghua Qiu

**Affiliations:** 1 Jiangxi Province Key Laboratory of Animal Nutrition and Feed, College of Animal Science and Technology, Jiangxi Agricultural University, Nanchang, China

**Keywords:** Antioxidant Capacity, Autoinducer-2, D-ribose, Microbial Density, Oxidative Stress, Quorum Sensing

## Abstract

**Objective:**

The objective of this study was to investigate the effects of dietary D-ribose supplementation on the growth performance, nutrient digestibility, serum biochemistry, rumen fermentation characteristics, and microbial LuxS/autoinducer-2 (AI-2) quorum sensing in Hu sheep.

**Methods:**

Eighteen female Hu sheep, aged 3 months with similar body weights (20.47±0.58 kg), were randomly divided into two groups of nine animals each. The control group was fed a basal diet (CON), while the experimental group received the basal diet supplemented with 300 mg/kg of D-ribose (DR). After 80 days of individual pen feeding, blood, rumen fluid, and fecal samples were collected for analysis.

**Results:**

The results showed that the average daily gain was higher in the DR group than in the CON group, and the feed-to-gain ratio was lower in the DR group (p<0.05). The apparent digestibility of ether extract tended to be higher in the DR group (p = 0.068). In comparison to the CON group, the levels of serum cortisol, malondialdehyde, and reactive oxygen species were lower in the DR group, while superoxide dismutase and glutathione peroxidase levels were higher, resulting in a decreased oxidative stress index (p<0.05). The rumen pH and microbial protein concentration were higher in the DR group (p<0.05), and no differences in ammonia nitrogen and volatile fatty acids concentrations were observed between the CON and DR groups (p>0.10). The rumen microbial density was higher in the DR group, while the concentrations of AI-2 signaling molecule, biofilm formation, and exopolysaccharides were lower (p<0.05).

**Conclusion:**

The findings of this study indicate that dietary supplementation with D-ribose can enhance the growth performance, improve serum antioxidant capacity, and inhibit rumen microbial LuxS/AI-2 quorum sensing in Hu sheep.

## INTRODUCTION

Ruminants are characterized by their distinctive digestive system, with the rumen as a key component. The rumen functions as a fermentation vessel, which harbors a diverse and active community of microorganisms, including bacteria, protozoa, fungi, and archaea. These microbes collaborate in degrading complex plant materials, such as cellulose and hemicellulose, into simpler compounds like volatile fatty acids (VFA), which serve as a primary energy source for ruminants. Additionally, the rumen microbes contribute to the production of microbial protein (MCP), an important nitrogen source for the host animal [1]. This complex symbiosis between ruminants and their rumen microbes is crucial for their survival and forms the basis of their productivity. The rumen microbiome’s impact extends beyond digestion, significantly affecting the overall health and performance of ruminants. It influences feed efficiency, growth rate, milk production, and even meat quality [2]. Maintaining the balance and activity of rumen microbes is essential for optimal rumen function and preventing disorders like acidosis and bloat [3]. Given the central role of rumen microbes in ruminant nutrition and health, manipulating the rumen microbiome has become a highly promising research area.

Feed additives have gained significant attention as an effective way to influence the rumen microbiome, thereby enhancing rumen fermentation and improving overall animal performance. Common feed additives, including probiotics, enzyme preparations, and plant extracts, have demonstrated their efficacy in enhancing rumen function. For instance, supplements of *Saccharomyces cerevisiae* can promote the growth of rumen fiber-degrading microbes and improve the efficiency of cellulose degradation [4,5]. Additionally, garlic extract can promote the growth of energy-producing bacteria, such as *Selenomonas ruminantium*, and denitrifying bacteria, such as *Pseudomonas stutzeri*, thereby facilitating the conversion of urea nitrogen into MCP and enhancing the utilization rate of urea nitrogen [6]. These findings highlight the potential benefits of feed additives to optimize rumen function and enhance the sustainability and efficiency of ruminant production systems.

As a natural five-carbon sugar and a key component of adenosine triphosphate (ATP), D-ribose plays a critical role in cellular energy production. It is especially important for sustaining energy levels in tissues with high metabolic needs, like the heart and skeletal muscles [7]. Among humans, D-ribose is gaining more attention due to its ability to boost cardiovascular performance, reduce fatigue, and enhance general productivity and wellness [8]. D-ribose has garnered increasing interest as a potential modulator of quorum sensing (QS), a microbial communication system that coordinates behavior based on population density [9]. This mechanism is vital for biofilm formation and the expression of virulence factors in numerous pathogens [9]. Recent research indicated that D-ribose, which shared structural similarities with the QS signaling molecule autoinducer-2 (AI-2), can inhibit biofilm development and suppress virulence gene expression by interfering with AI-2 receptors [10]. Harnessing the regulatory impact of D-ribose on QS could yield several advantages, including enhanced gut health, reduced pathogenic infections, and improved productivity and welfare [11]. Cai et al [12] revealed that enriching the diet of gibel carp (*Carassius auratus gibelio*) with D-ribose can substantially elevate their overall protein content levels and augment collagen and glycogen levels in muscle tissue. These enhancements, in turn, optimize the fish meat’s texture, water retention, and flavor compound accumulation. In horses, D-ribose supplementation has been proven effective in significantly bolstering muscle recovery following intense exercise [13]. However, the application of D-ribose in livestock production remains sparse in documentation, and its potential to influence the rumen microbiota in ruminants has yet to be explored.

Therefore, a 80-day feeding experiment was conducted to examine how D-ribose impacts growth performance, serum biochemistry, nutrient digestion, rumen fermentation, and microbial QS in Hu sheep. It was hypothesized that D-ribose could improve rumen fermentation and nutrient digestion by inhibiting the LuxS/AI-2 QS system of rumen microorganisms. This study is expected to provide a theoretical foundation for using D-ribose as a QS modulator to promote ruminant growth. Additionally, it is anticipated to advance the further development and research of rumen modulators for ruminants based on QS mechanisms.

## MATERIALS AND METHODS

### Diet, animals, and experimental design

A total of 18 healthy female Hu sheep, aged 3 months and with similar body weights (20.47±0.58 kg), were randomly divided into two groups, each consisting of nine sheep. The control group was fed a basal diet (CON), while the treatment group received the basal diet supplemented with 300 mg/kg of D-ribose (DR). The purity of D-ribose was 99%. The composition and nutritional components of the feed ingredients in the basal diet are detailed in Table 1. The feeding trial spanned 80 days, comprising a 20-day pre-feeding period and a 60-day feeding period. Throughout the trial, each animal was individually housed to ensure optimal living conditions, with the pens measuring 2.90 m in length and 2.30 m in width. They were provided with unrestricted access to feed and were consistently provided with clean, ample drinking water. The feeding schedule was meticulously maintained, with meals administered twice daily at 8:00 am and 6:00 pm. Notably, throughout the trial, none of the animals exhibited any signs of illness, and all maintained health.

### Sample collection

Daily records were maintained for feed intake and leftovers. The body weight of each sheep was measured on two consecutive days at both the start and end of the trial, with the average value serving as the initial or final body weight. Seven days before the end of the trial, fecal samples were collected from the rectum every six hours. Specifically, sampling was conducted at 0:00, 6:00, 12:00, and 18:00 on days 1, 3, 5, and 7, and at 3:00, 9:00, 15:00, and 21:00 on days 2, 4, and 6. This sampling method ensured that at least three days of samples with complete 3-hour intervals were collected, resulting in 28 sampling points per sheep. The total fecal output was estimated using acid-insoluble ash (AIA) as an internal marker [14]. Blood samples were collected from the jugular vein before morning feeding on two consecutive days before the end of the trial. After centrifugation of the blood samples at 4,000×g, serum was obtained. Similarly, on the last two days of the trial, rumen contents, including both liquid and solid phases, were collected before morning feeding using an oral cannula, as described by Paz et al [15]. After the rumen pH was measured, the contents were filtered through four layers of gauze to obtain the rumen fluid, which was then transferred to a −80°C laboratory freezer for storage.

### Parameter measurement

The indicators used to measure growth performance in this trial included average dry matter intake (DMI), average daily gain (ADG), and feed-to-gain ratio (F/G). The ADG was calculated by dividing the difference in body weight between the start and end of the trial by the number of feeding days. The F/G was determined by dividing the DMI by the ADG, with lower values indicating higher feed efficiency. The nutritional components in feed and feces, including dry matter, crude protein, ether extract, crude ash, starch, calcium, and phosphorus, were measured according to the corresponding methods in the AOAC guidelines [16]. Neutral detergent fiber (NDF) and acid detergent fiber (ADF) were measured using the method described by Van Soest et al [17], with thermostable *α*-amylase added during the extraction process. Apparent digestibility, expressed as a percentage, was calculated as the ratio of the difference between total feed intake and total fecal excretion to the total feed intake.

Serum cortisol was used to assess the stress levels of the animals, while immunoglobulin G (IgG) was used to evaluate their immune capacity. Antioxidant indicators included superoxide dismutase (SOD), glutathione peroxidase (GSH-Px), total antioxidant capacity (T-AOC), reactive oxygen species (ROS), and malondialdehyde (MDA). The oxidative stress index (OSI) was used to comprehensively evaluate the oxidative stress levels of the animals, and it was calculated as ROS divided by T-AOC. The reagent kits used for the detection of these indicators were purchased from the Beijing Sino-UK Institute of Biological Technology, and the guidelines provided with the reagent kits were strictly followed during the analysis.

Rumen fermentation characteristics were assessed by measuring rumen pH, ammonia nitrogen (NH_3_-N), MCP, and VFA. Rumen pH was promptly measured using a portable pH meter (testo 206; Testo) immediately after collection of rumen content. The concentrations of NH_3_-N and MCP were determined using the phenol-hypochlorite method [18] and Folin-phenol reagent colorimetric method [19], respectively. Both colorimetric analyses were conducted using a spectrophotometer (MK3; Thermo Fisher Scientific). The VFA, comprising acetate, propionate, isobutyrate, butyrate, isovalerate, and valerate, were identified by comparing their relative retention times to those of standards under identical conditions, and their concentrations were calculated based on relative peak areas. VFA analysis was performed using an Agilent 8860 gas chromatograph (Agilent Technologies) equipped with a 30 m HP INNOWAX capillary column (19091N-2131, 0.32 mm inner diameter×0.50 μm film thickness) and a flame ionization detector (FID-2019; Agilent Technologies), with nitrogen as the carrier gas. The parameter settings and operating procedures were consistent with those detailed by Luo et al [20].

The rumen microbial LuxS/AI-2 QS indicators included microbial density, AI-2 signaling molecule concentration, biofilm formation, and exopolysaccharide content. Initially, the rumen fluid is centrifuged at 1,000×g for 5 minutes to precipitate feed residues. The absorbance OD1 is then measured at 595 nm using the resultant supernatant. Subsequently, this supernatant is centrifuged at 12,000×g for 10 minutes, and the absorbance OD2 is measured at 595 nm using the supernatant obtained from this second centrifugation [21]. In parallel, distilled water is used as the blank control to measure the absorbance OD0 at the same wavelength of 595 nm. Finally, the microbial density is calculated using the formula OD1–OD2–OD0. The concentration of AI-2 signaling molecule was determined using the Fe(III)-1,10-phenanthroline method as described by Wattanavanitchakorn et al [22]. Biofilm formation was evaluated using the crystal violet method, and the exopolysaccharide content was quantified using the phenol-sulfuric acid method, with glucose as the standard [21].

### Statistical analysis

Initially, the Shapiro-Wilk test was employed to examine the normality of all data sets. Since the data conformed to a normal distribution (p>0.05), a t-test was used to compare the CON group and the DR groups. The analysis was conducted using SPSS (ver. 20; IBM). A p-value of less than 0.05 (p<0.05) was considered to indicate a statistically significant difference, while a p-value between 0.05 and 0.10 (0.05≤p<0.10) was taken as an indication of a potential trend.

## RESULTS

### Growth performance

The effect of dietary supplementation with D-ribose on growth performance of Hu sheep is shown in Table 2. The DR group had higher ADG and lower F/G compared to the CON group (p<0.05), while no significant difference was observed in average DMI between the CON and DR groups (p>0.10).

### Nutrient apparent digestibility

The effect of dietary supplementation with D-ribose on nutrient apparent digestibility of Hu sheep is listed in Table 3. The apparent digestibility of ether extract in the DR group tended to be higher than that in the CON group (p = 0.068), while no significant differences were observed in the apparent digestibility of other nutrients between the CON and DR groups (p>0.10).

### Serum biochemical indices and antioxidant capacity

The effects of dietary supplementation with D-ribose on serum biochemical indices and antioxidant capacity of Hu sheep are detailed in Table 4. The cortisol concentration in the DR group was lower than that in the CON group (p<0.05), while no significant difference was observed in IgG content between the CON and DR groups (p>0.10). Supplementation with D-ribose reduced the levels of MDA and ROS, while increasing the levels of SOD and GSH-Px, ultimately leading to a decreased OSI (p<0.05).

### Rumen fermentation characteristics

The effect of dietary supplementation with D-ribose on rumen fermentation characteristics of Hu sheep is presented in Table 5. The rumen pH and MCP concentration in the DR group were higher than those of the CON group (p<0.05). Dietary supplementation with D-ribose had no effect on the total VFA concentration, nor did it significantly influence the proportions of individual VFA (p>0.10).

### Rumen bacterial quorum sensing

**Figure 1:**
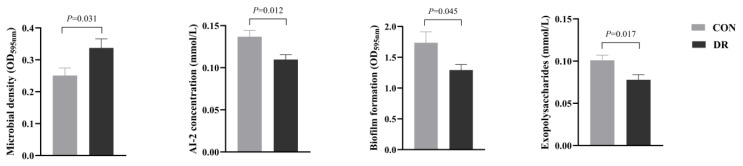
Rumen microbial LuxS/autoinducer-2 (AI-2) quorum sensing of Hu sheep fed a basal diet (CON) and a basal diet supplemented with 300 mg/kg of D-ribose (DR).

As shown in Figure 1, the microbial density in the DR group was higher than that in the CON group (p<0.05), while the concentration of AI-2 signaling molecule, the amount of biofilm formation, and the content of exopolysaccharides were significantly lower in the DR group than in the CON group (p<0.05).

## DISCUSSION

ATP is recognized as the principal source of energy within cells, with D-ribose acting as a crucial precursor for ATP synthesis. Supplementing with D-ribose has been shown to increase ATP production, reduce cellular damage, and stimulate energy metabolism [23]. This not only elevates overall energy levels but also facilitates the swift recovery and repair of muscle tissue damaged by physical activity in humans, thereby enhancing their physical performance capabilities [24]. This study found that, given equivalent levels of average DMI, the DR group achieved superior ADG and better feed efficiency. Moreover, the addition of D-ribose to the diet has been observed to marginally enhance the digestibility of ether extract. Although the enhancements in the digestibility of other nutrients did not reach statistical significance, a consistent pattern of numerical improvement was observed across all of them.

The adrenal cortex is responsible for secreting cortisol, a glucocorticoid hormone that is critical for managing stress. When the body experiences stress, the hypothalamus releases corticotropin-releasing hormone (CRH), which stimulates the anterior pituitary gland to secrete adrenocorticotropic hormone (ACTH). This hormonal cascade ultimately results in the production of cortisol by the adrenal cortex. Consequently, cortisol concentrations serve as a barometer for stress levels and the body’s resilience to environmental stressors [25]. In this study, the DR group exhibited significantly reduced cortisol levels compared to the control group, suggesting that D-ribose supplementation could potentially mitigate stress. SOD and GSH-Px are two vital antioxidant enzymes that play crucial roles in safeguarding cellular structures and functions. SOD effectively neutralizes superoxide radicals, thereby protecting cell membranes, proteins, and DNA from oxidative damage [26]. Meanwhile, GSH-Px combats oxidative stress by detoxifying hydrogen peroxide and other peroxides, thus preventing the accumulation of harmful oxidative byproducts within cells and maintaining cellular integrity [27]. In contrast, ROS and MDA are key contributors to the development and progression of various diseases. ROS, produced during cellular metabolism, are highly reactive molecules with strong oxidative properties. When their concentration exceeds the capacity of the cell’s antioxidant mechanisms, they trigger oxidative stress, which can harm cellular biomolecules [28]. MDA, by contrast, is a byproduct of lipid peroxidation. Higher levels of MDA usually indicate that lipid peroxidation has occurred within the cell [29]. In this study, the DR group exhibited increased levels of SOD and GSH-Px, along with decreased levels of ROS and MDA. This indicates that D-ribose supplementation effectively reduces the stress level of Hu sheep, a conclusion further supported by the reduced oxidative stress index and cortisol level.

Ruminal pH is a critical environmental factor essential for the survival and activity of rumen microorganisms, directly impacting the health and productivity of ruminants. Both excessively high and low ruminal pH levels can impair microbial function, thereby disrupting normal rumen fermentation and reducing the digestibility of key dietary components like fiber and protein [30]. Additionally, extreme fluctuations in ruminal pH can lead to serious health issues, such as ruminal acidosis, characterized by a significant drop in pH due to organic acids accumulation [31]. These effects can negatively impact overall health, resulting in slower growth, decreased milk production, and increased disease susceptibility. In this study, dietary D-ribose supplementation elevated ruminal pH, which remained within the normal range. The observed elevation in ruminal pH could be driven by multiple factors. One likely reason is the numerically reduced concentration of VFA. As end products of microbial fermentation in the rumen, VFA can cause a drop in ruminal pH when they accumulate [31]. Therefore, a decline in VFA concentration may lead to an increase in ruminal pH. Nevertheless, it should be emphasized that ruminal pH is influenced by a complex interplay of various factors. These include the composition of the microbial community, the type and quality of feed, saliva production, and the physiological state of the animal [32]. The impact of D-ribose on ruminal pH is likely a result of the combined effects of these factors, rather than a single cause. Integrating genomic and metabolomic analyses will offer a more comprehensive understanding of how D-ribose supplementation increases ruminal pH. MCP is a vital source of metabolizable protein for ruminants. It is synthesized through the assimilation of ammonia and the formation of carbon skeletons, which convert nitrogen sources in the feed into high-quality proteins [33]. Under the same dietary feeding regimen, a higher value of MCP indicates a greater utilization rate of nitrogen sources by ruminants. The fact that the MCP in the DR group was higher than that in the control group suggests that the addition of D-ribose can enhance the utilization of nitrogen sources in the diet. This effect may be related to the inherent ability of D-ribose to boost energy metabolism of both microbial cells and host, which can be indirectly inferred from the numerically lower concentration of NH_3_-N, higher MCP, and the trend of increased apparent digestibility of ether extract observed in the DR group. However, it remains to be explored whether D-ribose can be absorbed across the rumen wall to enhance the host’s energy metabolism.

QS serves as a vital communication mechanism among bacteria, enabling microbes to coordinate their collective behaviors through the secretion and detection of signaling molecules [34]. This process plays a crucial role in modulating microbial metabolic activities and ecological functions. Research has identified AI-2 as the predominant signaling molecule utilized by rumen microbes for communication, influencing key aspects such as biofilm formation, fermentation processes, and nutrient utilization efficiency [35,36]. Studies have further demonstrated that abrupt shifts in dietary composition, fluctuations in rumen drinking water temperature, and the introduction of feed additives can all trigger responses within the LuxS/AI-2 QS system of rumen microbes [37–39]. These responses help regulate microbial stress resistance and facilitate adaptation to changing environmental conditions. The rumen microbial density serves as a reflection of the richness and activity within the rumen microbial community. A higher microbial density generally signifies that the microbes are capable of efficiently decomposing and utilizing the nutrients present in the feed [2]. Additionally, it suggests that the microbial community as a whole possesses a greater degree of stability. In the context of this study, the DR group was observed to have a higher microbial density, which aligns with the numerically increased nutrient digestibility seen in this group. This finding indicates that the D-ribose supplementation has the potential to enhance the stability and richness of the rumen microbial community, ultimately leading to improved nutrient digestion. Interestingly, the D-ribose supplementation was found to reduce the levels of the AI-2 signaling molecule, which is involved in QS among rumen bacteria. This reduction also extended to biofilm formation and its primary component, exopolysaccharides. The likely explanation for this phenomenon is that D-ribose functions as a LuxS/AI-2 QS inhibitor. By interfering with the AI-2 signaling molecule, it can effectively inhibit the expression of virulence genes and the formation of biofilms in pathogenic bacteria [9]. This action, in turn, enhances the host’s immune and antioxidant capabilities, which can be indirectly inferred from the lower stress levels and higher antioxidant capacity observed in the DR group. Despite the inhibitory effect of D-ribose on LuxS/AI-2 QS, the microbial density still increased. This can be attributed to the fact that the rumen microbial community is a complex mixture of various microorganisms, including bacteria, archaea, protozoa, and fungi [34]. D-ribose may specifically targets and inhibits LuxS/AI-2 QS in rumen pathogenic bacteria without affecting other types of microbes. However, this hypothesis needs to be verified through a comprehensive analysis of the diversity and community composition of rumen microbiota. Moreover, it is worth noting that other communication mechanisms involving different signaling molecules, such as N-acyl-homoserine lactones (AHL) and autoinducing peptides (AIP), may also be at play in QS among rumen bacteria [34,36]. Given these findings, future research may explore the impact of D-ribose on diverse signaling molecules across various rumen microbial species. Such investigations will provide a more comprehensive understanding of how D-ribose enhances ruminant growth, as viewed through the lens of QS.

## CONCLUSION

Taken together, dietary supplementation with 300 mg/kg of D-ribose enhanced the ADG and feed efficiency of Hu sheep. The DR group tended to increase the apparent digestibility of ether extract while reducing the levels of serum cortisol, MDA, and ROS, as well as the oxidative stress index, compared to the CON group. Additionally, the D-ribose supplementation elevated the levels of serum SOD and GSH-Px. The addition of D-ribose increased the rumen microbial density but decreased the levels of the AI-2 signaling molecule, biofilm formation, and exopolysaccharides. These results suggest that dietary supplementation with D-ribose can improve growth performance, enhance serum antioxidant capacity, and inhibit rumen microbial LuxS/AI-2 QS of Hu sheep. The findings of this study may provide new insights into the application of QS modulators in enhancing ruminant production.

## Figures and Tables

**Table 1:** Feed ingredients and chemical composition of the basal diet

Ingredient	Proportion (%)	Chemical composition	Value
Corn	25.00	Metabolizable energy (Mcal/kg)	2.40
Soybean meal	10.50	Crude protein (g/kg)	130.61
Wheat bran	12.75	Neutral detergent fiber (g/kg)	402.03
Wheat straw	28.75	Acid detergent fiber (g/kg)	245.58
Peanut straw	20.00	Ether extract (g/kg)	26.65
Calcium hydrogen phosphate	1.00	Calcium (g/kg)	8.66
Limestone	0.50	Phosphorus (g/kg)	4.98
Salt	0.50		
Premix1)	1.00		

1) Premix provided the following per kg of DM: 1,400 mg of Fe, 1,200 mg of Zn, 250 mg of Cu, 900 mg of Mn, 100,000 IU of vitamin A, 27,000 IU of vitamin D3, and 800 IU of vitamin E.

**Table 2:** Effect of dietary supplementation with D-ribose on growth performance of Hu sheep

Item	CON	DR	SEM	p-value
Initial body weight (kg)	20.48	20.46	0.842	0.985
Final body weight (kg)	28.49	29.82	1.046	0.383
Average daily gain (g/d)	133.52	156.02	6.700	0.032
Average dry matter intake (g/d)	900.08	893.34	14.185	0.959
Feed-to-gain ratio	6.92	5.79	0.314	0.027

CON, control, basal diet; DR, basal diet supplemented with 300 mg/kg of D-ribose; SEM, standard error of the mean.

**Table 3:** Effect of dietary supplementation with D-ribose on nutrient apparent digestibility (%) of Hu sheep

Item	CON	DR	SEM	p-value
Dry matter	60.55	62.94	1.309	0.392
Crude protein	66.39	69.25	1.161	0.209
Ether extract	52.34	56.00	1.210	0.068
Acid detergent fiber	38.17	45.88	3.017	0.179
Neutral detergent fiber	38.06	45.50	2.725	0.190
Crush ash	63.31	62.61	1.113	0.558
Starch	48.33	55.24	2.729	0.186
Calcium	32.96	37.02	2.244	0.306
Phosphorus	29.58	43.13	4.137	0.148

CON, control, basal diet; DR, basal diet supplemented with 300 mg/kg of D-ribose; SEM, standard error of the mean.

**Table 4:** Effects of dietary supplementation with D-ribose on serum biochemical indices and antioxidant capacity of Hu sheep

Item	CON	DR	SEM	p-value
Cortisol (ng/mL)	50.07	31.32	1.100	<0.001
Immunoglobulin G (g/L)	16.63	17.40	1.009	0.611
Malondialdehyde (nmol/mL)	4.93	3.57	0.226	0.001
Reactive oxygen species (U/mL)	277.37	137.80	7.638	<0.001
Superoxide dismutase (U/mL)	67.11	84.87	1.623	<0.001
Glutathione peroxidase (U/mL)	238.08	335.48	5.053	<0.001
Total antioxidant capacity (U/mL)	9.66	10.12	0.426	0.473
Oxidative stress index	28.89	13.84	1.044	<0.001

CON, control, basal diet; DR, basal diet supplemented with 300 mg/kg of D-ribose; SEM, standard error of the mean.

**Table 5:** Effect of dietary supplementation with D-ribose on rumen fermentation characteristics of Hu sheep

Item	CON	DR	SEM	p-value
Rumen pH	6.51	6.76	0.057	0.007
Ammonia nitrogen (mg/dL)	9.99	8.09	1.151	0.261
Microbial protein (μg/mL)	1,218.54	1,629.72	66.133	0.001
Concentration (mmol/L)				
Acetate	30.61	27.30	1.643	0.183
Propionate	10.64	11.47	0.653	0.384
Isobutyrate	0.47	0.49	0.061	0.780
Butyrate	5.68	5.43	0.779	0.831
Isovalerate	0.90	0.81	0.146	0.647
Valerate	0.69	0.71	0.068	0.839
Total volatile fatty acids	48.99	46.20	2.066	0.378
Branched-chain volatile fatty acids	2.06	2.01	0.174	0.841
Proportion (%)				
Acetate	62.30	59.02	1.796	0.216
Propionate	22.23	24.99	1.651	0.258
Acetate to propionate ratio	2.99	2.45	0.251	0.156
Isobutyrate	0.95	1.06	0.123	0.531
Butyrate	11.24	11.64	1.327	0.841
Isovalerate	1.82	1.73	0.299	0.853
Valerate	1.47	1.56	0.178	0.739
Branched-chain volatile fatty acids	4.24	4.35	0.342	0.816

CON, control, basal diet; DR, basal diet supplemented with 300 mg/kg of D-ribose; SEM, standard error of the mean.

## Data Availability

Upon reasonable request, the datasets of this study can be available from the corresponding author.
